# Feasibility of forecasting future critical care bed availability using bed management data

**DOI:** 10.1136/bmjhci-2024-101096

**Published:** 2024-08-19

**Authors:** John Palmer, Areti Manataki, Laura Moss, Aileen Neilson, Tsz-Yan Milly Lo

**Affiliations:** 1 Center for Medical Informatics, The University of Edinburgh Usher Institute of Population Health Sciences and Informatics, Edinburgh, UK; 2 School of Computer Science, University of St Andrews, St Andrews, UK; 3 Department of Clinical Physics and Bioengineering, NHS Greater Glasgow and Clyde, Glasgow, UK; 4 School of Medicine, University of Glasgow, Glasgow, UK; 5 Edinburgh Clinical Trials Unit, The University of Edinburgh Usher Institute of Population Health Sciences and Informatics, Edinburgh, UK; 6 Paediatric Critical Care Unit, Royal Hospital for Children and Young People, Edinburgh, UK

**Keywords:** Machine Learning, Computing Methodologies, Data Science, Decision Support Systems, Management

## Abstract

**Objectives:**

This project aims to determine the feasibility of predicting future critical care bed availability using data-driven computational forecast modelling and routinely collected hospital bed management data.

**Methods:**

In this proof-of-concept, single-centre data informatics feasibility study, regression-based and classification data science techniques were applied retrospectively to prospectively collect routine hospital-wide bed management data to forecast critical care bed capacity. The availability of at least one critical care bed was forecasted using a forecast horizon of 1, 7 and 14 days in advance.

**Results:**

We demonstrated for the first time the feasibility of forecasting critical care bed capacity without requiring detailed patient-level data using only routinely collected hospital bed management data and interpretable models. Predictive performance for bed availability 1 day in the future was better than 14 days (mean absolute error 1.33 vs 1.61 and area under the curve 0.78 vs 0.73, respectively). By analysing feature importance, we demonstrated that the models relied mainly on critical care and temporal data rather than data from other wards in the hospital.

**Discussion:**

Our data-driven forecasting tool only required hospital bed management data to forecast critical care bed availability. This novel approach means no patient-sensitive data are required in the modelling and warrants further work to refine this approach in future bed availability forecast in other hospital wards.

**Conclusions:**

Data-driven critical care bed availability prediction was possible. Further investigations into its utility in multicentre critical care settings or in other clinical settings are warranted.

**WHAT IS ALREADY KNOWN ON THIS TOPIC**

- Bed capacity forecasts have been successfully applied in the emergency department and at a whole hospital level using temporal trends in bed occupancy. In critical care, methods predicting individual patient length of stay have previously been developed which require sensitive clinical data, however, no prior work has been conducted modelling multivariate temporal trends in whole hospital bed management data to forecast future bed capacity in critical care.

**WHAT THIS STUDY ADDS**

- We demonstrated for the first time that routinely collected hospital bed management data could be used to predict future critical care bed capacity using time series modelling methods. Temporal trends in resource information such as arrivals, transfers and discharges could improve forecasting performance. It also demonstrated a method for classifying whether at least one critical care bed will be available using this routinely collected non-sensitive data.

**HOW THIS STUDY MIGHT AFFECT RESEARCH, PRACTICE OR POLICY**

- Successfully demonstrating the feasibility of using a data-driven tool to predict future critical bed availability in this study drives future research validation on how this tool may actually enable improved hospital resource planning (eg, staff and elective admission scheduling within critical care). Furthermore, our methodology developed in this study may be readily transferable to predicting bed availability in other centres and different clinical settings with routinely collected hospital bed management/capacity data.

## Background

 Intensive care units (ICU) are often operating at, or very close to, full capacity.^1 2^ With this high demand for ICU beds, elective admissions often require rescheduling to a later date at short notice with emergency/unplanned admissions taking priority. Late rescheduling of elective cases causes stress and uncertainty for patients and their families as well as wasted hospital resources.^3^ Being able to accurately predict if any ICU beds would be available on a future day would enable key stakeholders in healthcare systems to better plan for ICU elective admissions with a lower risk of cancellation and more efficient staff scheduling.^4^

Previous researchers have attempted to directly predict bed availability at an individual patient level or at a higher aggregated level.^5^,^8^ Other methods use individual patient length of stay (LoS) data to estimate whether a bed will be available on a future day. This type of modelling requires accurate prediction of patient LoS^9^,^13^ coupled with estimations of arrival^14^,^16^ and discharge volume^17^ to enable bed availability forecasts over short prediction horizons.^18^ However, these approaches require access to detailed individual patient data including clinical, demographic and physiological data. This is very labour-intensive to extract beyond research settings. Furthermore, the models used to predict LoS are also often complex with large numbers of parameters leading to problems of prediction explainability, which may impair trust among their intended end users (clinicians and clinical management teams). A simpler approach using readily available hospital bed management data (ie, temporal trends in overall unit admissions, discharges and occupancy levels) to predict ICU bed capacity would, therefore, be highly desirable. The advantages of this approach include (1) utilisation of routinely collected resource data that do not require complex data extraction, (2) no complex LoS models that require detailed patient-level data and (3) models which are simpler to evaluate and understand. To the best of the authors’ knowledge, no prior research has used hospital-wide bed management data to predict bed availability in the ICU.

This project aims to use a single-centre paediatric ICU (PICU) as a case study to determine if (1) it is feasible to predict whether a critical care bed is available using only the routinely collected bed management data (aim 1) and (2) the bed availability prediction may be made 1, 7 and 14 days in advance (aim 2).

## Methods

This study is a single-centre data informatics study using fully anonymised, prospectively collected, routine hospital bed management data generated from clinical care (between 2012 and 2019) at one Scottish tertiary referral hospital. All analyses were performed retrospectively (offline). This project study is registered as a hospital quality improvement project.

**Figure 1:**
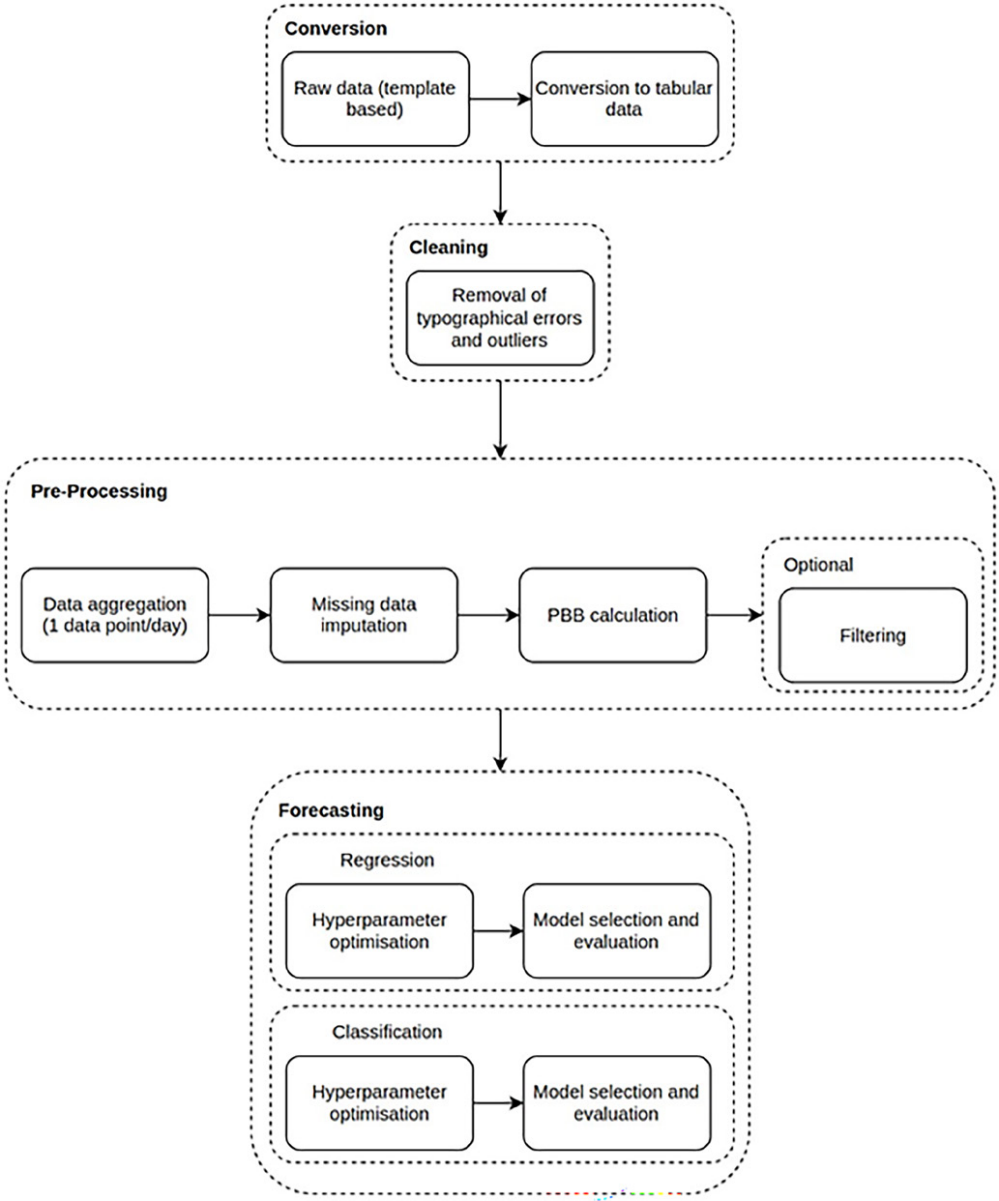
Data processing and modelling workflow. PBB, potential bed base.

The data contains features relating to the daily usage of beds for the PICU and other hospital wards. The data are collected either once or twice per day depending on the year of collection. Figure 1 summarises the overall methodology required to prepare the raw data for the forecasting analyses.

The raw routine bed management data were collected by manual input into a semistructured Microsoft Excel template, generating a completed data template for each time point over an 8-year period. Relevant information from each template was extracted and collated into a data table containing bed usage resource data for each department (including PICU) at a specific time point. Typographical errors with invalid values were corrected manually where possible while errors for which there was no obvious correction were deleted.

Only one data point was collected each day in the morning before 2014, while after this date, there were two data collection points each day in the morning and afternoon. Data post-2014 was, therefore, aggregated into a singular data point per day to produce a uniform sample frequency for our analyses.

Our data contained two different types of data features which required different preparations before analyses: (1) count-based data that is conditioned on the previous value which in this case was the number of available beds in the unit. The maximum value per day was taken for these data and (2) count-based data that is not conditioned on the previous values such as the number of admissions or discharges to the unit. For these data, the values for each day were summed as they were cumulative. Imputation was applied to fill missing data points to ensure a uniform frequency of data points. In case (1) data features were imputed using a forward fill methodology while in case (2) data features were imputed through sampling from a random variable inferred from the training data.

The final data set used for analyses consisted of 2906 data points with one data point per day. A total of 5.9% of the data were imputed.

The potential bed base (PBB) is a measure of the current bed state as well as the expected number of patients taking and leaving a bed in the next period. It is used by hospital management to ascertain whether there will be a bed available for a patient to be admitted. For this work, the PBB in the PICU is being modelled for forecasting. The PBB for a period is calculated using Equation 1 where **n** is the counts of the resource or events occurring in the given period. The available bed (and therefore the PBB also) can be negative when unfunded beds are used. For this work when the number of available beds has dropped below zero it is treated as there being no beds available.


(Equation 1) $$\begin{array}{ll} PBB= & n_{beds\;available}+n_{cubicles\;available}+n_{discharges}+\\ n_{transfers\;out}-n_{transfers\;in}-n_{admissions} \end{array}$$

Our time series data consisted of a low-frequency trend and a high-frequency component. The low-frequency component was extracted using a low pass bidirectional filter applied to the time series. As the level of smoothing can be difficult to identify, the filter order and cut-off frequency were adjusted as part of the hyperparameter experiments when assessing the best model.

### Forecasting techniques

We used two types of forecasting methods depending on the task being assessed. Regression-based methods were used to predict the absolute number of beds currently unoccupied in the unit (aim 1) while binary classification techniques were used to forecast whether there will be at least one free bed on a future date (aim 2).

For aim 1, two random walk models were used as a baseline. Random walk models use the last measured point projected over the forecasting horizon for prediction. One used the last measured value and projected this forward over the forecasting horizon, and the second used the smoothed value projected over the forecasting horizon. The parameterised regression models used in this work were ridge regression (L1 regularisation), lasso regression (L2 regularisation), Bayesian linear regression, XGBoost regression, D-linear model and long short-term memory (LSTM) models. Regression models were chosen as those with the best root mean absolute error (MAE) as compared with the actual (ground truth) validation data. All models are standard for use in time series forecasting problems.

For aim 2, we used logistic regression (with L1, L2 or elastinet regularisation), support vector machines with radial basis function or sigmoid kernel functions, XGBoost classifier, Gaussian naive Bayes and K-Nearest Neighbours. These methods are often used in binary classification problems and their usage is well understood. Due to the inherent class imbalance in the data (23.8% of the training data consisted of the class where there were no beds), the area under the curve (AUC) of the receiver operator characteristic curve score was used to assess the performance of the models. No baseline model was required as any classifier with AUC of above 0.5 is performing better than a classifier based solely on random chance.

In the simplest case, forecasting models were trained solely on a number of historical data points of the PICU PBB, however, forecasting models can also be trained using historical data points for related features. The incorporation of these features could improve the forecasting performance of the models if they contain useful information on the future PBB in the PICU across the prediction horizon. The features used in this work can be broadly broken down into two main categories: (1) hospital-related features and (2) temporal features. The hospital-related features include available beds, admissions, discharges and transfers from all of the units while the temporal features include the day of the year, day of the week, week, month and whether a day is a public and/or school holiday.^7 19^ All features were scaled to 0 mean and unit SD using the mean and SD calculated on the training data.

Hyperparameter optimisation was conducted. The data were split into a training/validation/test set in a 67.5%/22.5%/10% ratio retaining the temporal ordering. For each forecasting model, 250 experiments were conducted with random hyperparameters fitted on the training data. The forecasting model with the best performance with either the minimum MAE (aim 1) or maximum AUC (aim 2) was calculated using predictions on the validation data and the associated ground truth was selected.

## Results

Forecasting future bed availability using routinely collected hospital resource data was possible. Regression-based techniques enabled the forecasting of the underlying trends in the number of available beds. Classification techniques enabled the determination of whether at least one bed will be available on a future date. Prediction performance of our models was observed to worsen with increasing forecasting horizon.

**Figure 2:**
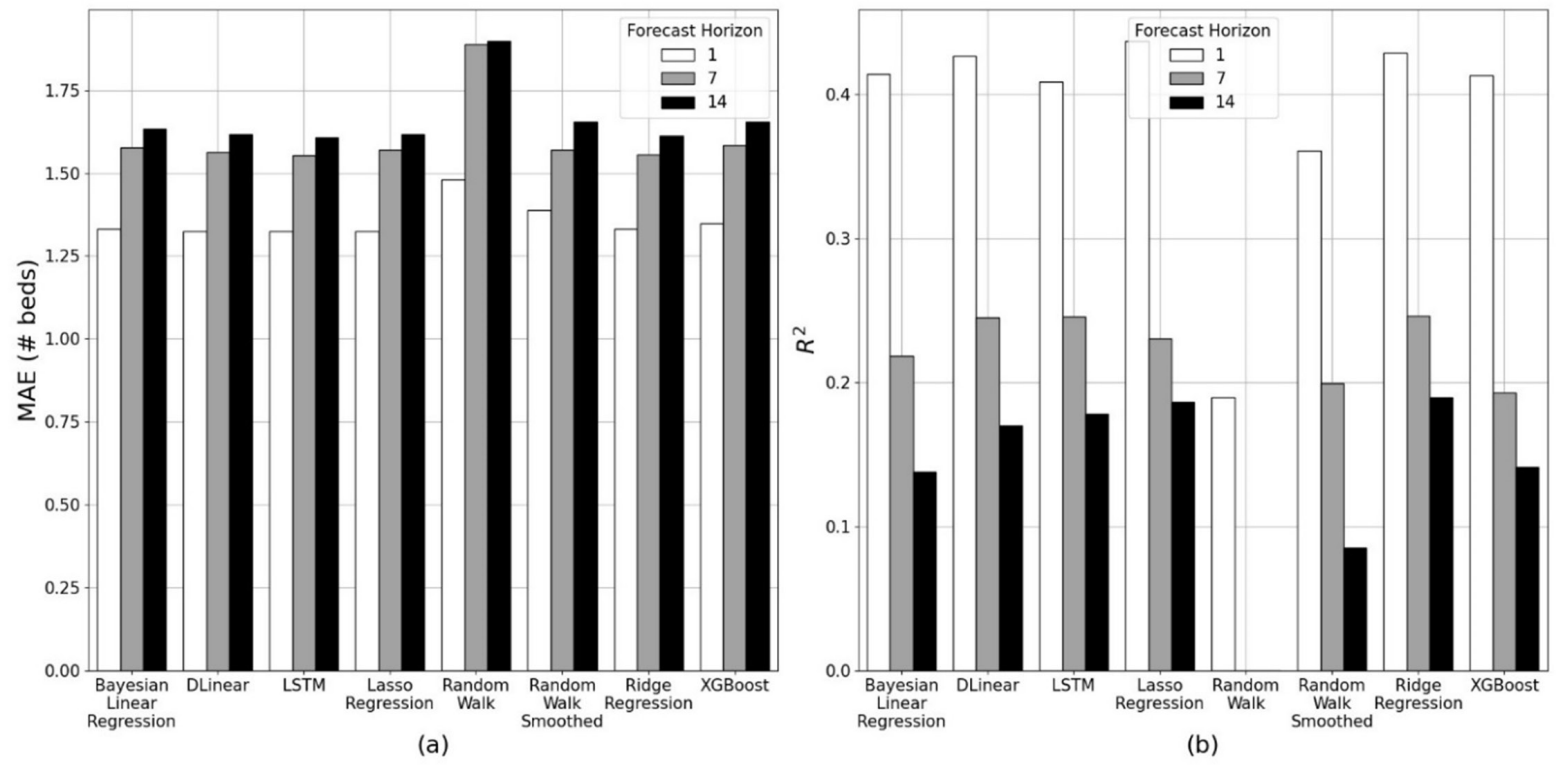
Regression results showing the (A) MAE and (B) R^2^ for the best-performing forecasting model of each type on the validation data over 1, 7 and 14 days forecasting horizons. The forecasting performance is improved over shorter forecasting horizons. LSTM, long short-term memory; MAE, mean absolute error.

Lasso regression, DLinear and LSTM models performed the best (figure 2) when predicting bed availability for the next day (MAE validation: 1.33, test: 1.31). For prediction 7 and 14 days in advance, ridge regression and LSTM performed equally the best over both forecasting horizons (MAE validation: 1.55, test: 1.48 and MAE validation: 1.61, test: 1.48, respectively). Over the longer forecasting horizons, the MAE converged towards that of the baseline smoothed random walk model. All parametric models performed better than the non-smoothed random walk model. In the case of equal model performance, the simpler model (ie, the linear models) was selected as the most suitable.

**Figure 3:**
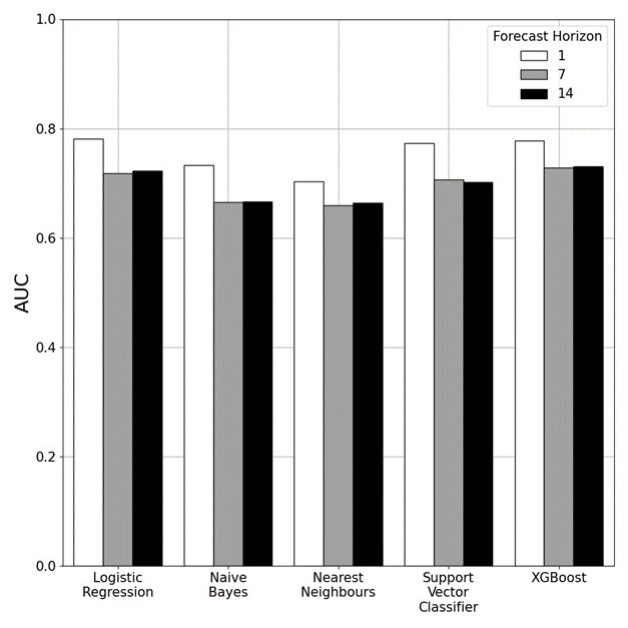
Classification results showing the AUC for the best-performing forecasting model of each type on the validation data over 1, 7 and 14 days forecasting horizons. The forecasting performance is improved over shorter forecasting horizons. AUC, area under the curve.

Figure 3 summarises the best results for each of the classification models across each forecasting horizon. Unlike the previous task where linear models performed the best or equal best over every forecasting window, logistic regression performed the best over a single day while over the longer forecasting horizons XGBoost models gave the best performance. Forecasting performance again worsened as the prediction window was increased from 1 day (AUC validation: 0.78, test: 0.75) to 7 days (AUC validation: 0.73, test: 0.66) and then 14 days (AUC validation: 0.73, test: 0.69).

## Discussion

This single-centre data informatics feasibility study demonstrated for the first time that forecasting ICU bed availability was possible using only routinely collected hospital bed management data without the need for detailed and sensitive clinical data. Our data-driven bed availability prediction performed better over shorter prediction time frames.

We are the first to present a novel reformulation to the bed occupancy problem by classifying the availability of one or more beds on a future date. This is an important distinction when trying to predict the exact number of available beds, as it is a simpler problem to solve. Although more limited in clinical scope, this formulation has specific applications when assessing if a bed will be available for upcoming admissions to the unit while also being easier to interpret and use. Having confirmed our model could forecast the availability of at least 1 PICU bed, the methodology could in the future be generalised to classify any number of available beds. For example, the forecasting of at least two ICU beds availability would account for having an emergency bed available if elective surgery planned for the day were to proceed. Forecasting ICU bed availability allows time for an elective surgery to be rescheduled and to reallocate resources thereby reducing wastage. Preventing last minute surgery cancellation may help to reduce distress for the patient and their family. Future clinical translation and implementation of any data-driven bed availability forecasting model will depend on achieving a forecasting accuracy or an AUC level that is of clinical importance. Currently, there is no agreed clinically significant accuracy or AUC level in the literature for these types of bed availability forecasting models, and this warrants future coinvestigations between clinicians and data scientists.

Using hospital bed management data to predict hospital-wide bed availability has been reported previously,^7 19 20^ but we are the first to use this approach to predict daily ICU bed capacity specifically. Previously, the prediction of future intensive care bed availability relies on detailed and sensitive clinical data.^21^ The majority of the previous work in bed availability prediction required, at the very least, detailed patient LoS data, for example, summary LoS statistics such as the mean^22 23^ or use techniques such as compartment modelling^24^ and discrete event simulation.^25 26^ Other approaches use detailed patient-level clinical, demographic and physiology data to predict individual remaining LoS.^18^ However, they could only predict short horizons into the future based on the LoS of patients in the unit with specific pathologies. Extracting and analysing detailed patient-level data are also challenging requiring complex ethical and regulatory considerations. Using state-of-the-art forecasting methods applied only to the temporal trends present in non-sensitive, non-patient-based routinely collected hospital management data to predict ICU bed capacity as we did in our project offered a clear advantage for wider uptake. This is because secondary analysis of routinely collected hospital bed management data can be performed by all hospitals in Britain as quality improvement initiatives.

For the classification task, Gradient boosted tree methods had the best performance over 7 and 14 days and logistic regression performed the best over 1-day prediction. Simpler linear models performed equally when compared with the complex non-linear methods in the regression task and were a surprising finding in our results, especially when other similar work in literature has moved towards non-linear neural network-based models. Kutafina *et al*^7^ used a recursive neural network-based model architecture while Kumar *et al*^20^ used a standard neural network with both showing improved results over older literature which used linear models.^19^ It is well known that recurrent neural network based models such as LSTMs are typically very suitable for long-term forecasting based on their ability to memorise key temporal patterns.^27^ In contrast, in our study, LSTMs performance only equalled the simpler linear models, as did other state-of-the-art forecasting models such as DLinear which automatically extract frequency-based information such as seasonality.^28^ It is possible that the propensity of these models to overfit the data coupled with the large number of available hyperparameters and the high relative variance observed in this dataset led to difficulty in finding an optimal model. This did, however, lead to models with a degree of explainability as it was possible to directly evaluate the impact of feature importance with both linear and XGBoost models.

**Figure 4:**
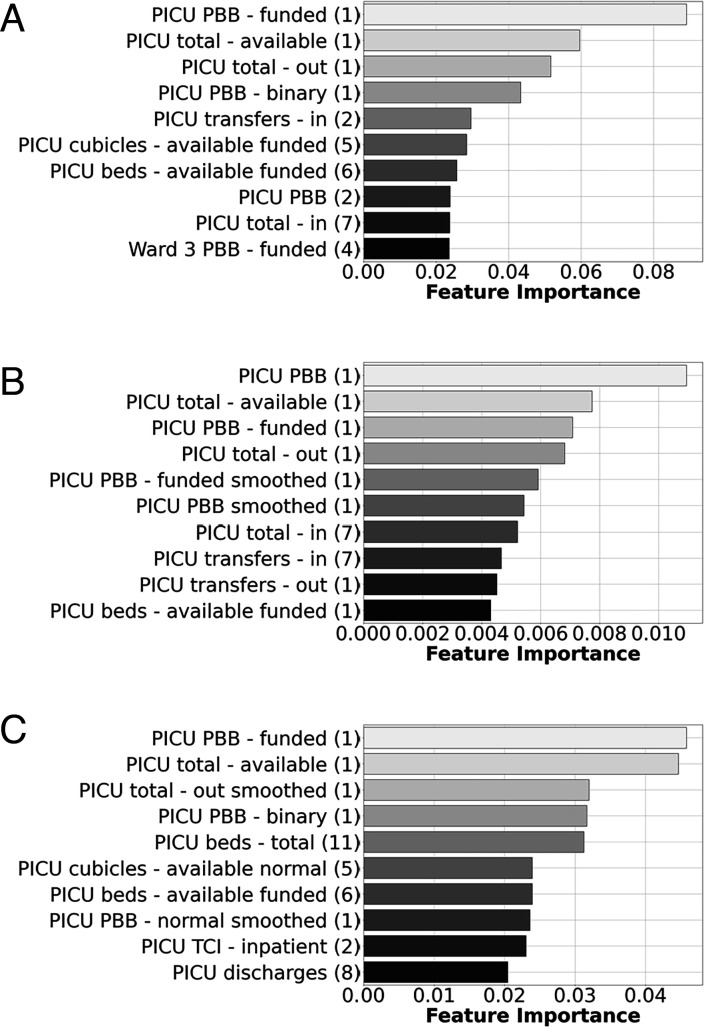
Feature importance showing the 10 features with the largest magnitude of top-performing regression models used to determine whether a PICU bed will be available in (A) 1 day, (B) 7 days and (C) 14 days. Features with larger magnitude weights have a bigger impact on the model predictions. Numbers in brackets indicate features lagged by that many days. PBB, potential bed base; PICU, paediatric intensive care unit; TCI, to come in.

**Figure 5:**
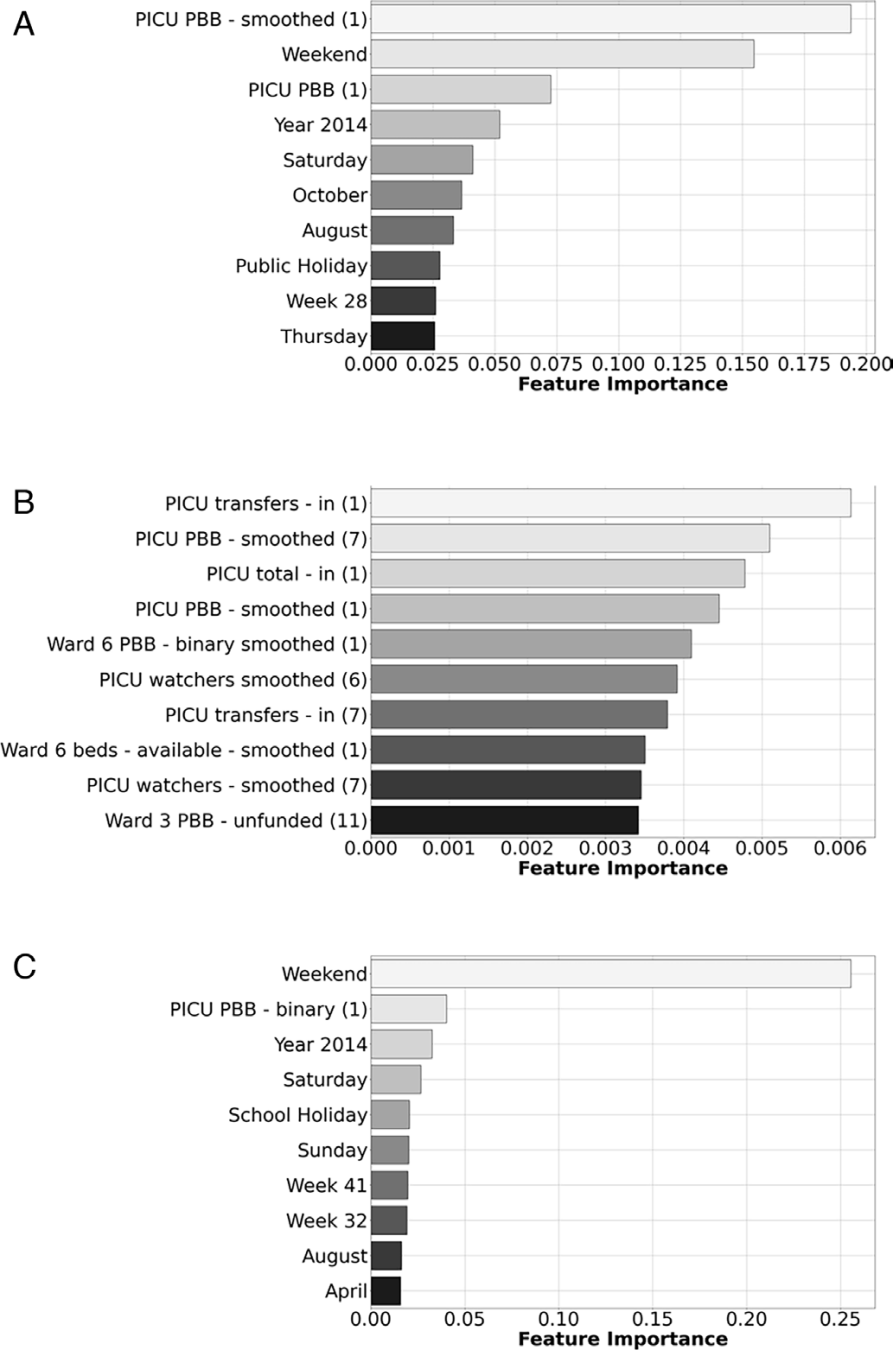
Feature importance showing the 10 features with the largest magnitude of top-performing logistic regression models used to determine whether a PICU bed will be available in (A) 1 day, (B) 7 days and (C) 14 days. Features with larger magnitude weights have a bigger impact on the model predictions. Numbers in brackets indicate features lagged by that many days, and the weeks reference the weeks of the year: for example, week 28 is a week in the Edinburgh school summer holidays, week 32 is the first week of Edinburgh Autumn school term while week 41 is the autumn half term week in October of Edinburgh school term. PBB, potential bed base; PICU, paediatric intensive care unit.

All the top-performing models in this project had access to features not only relating to previous PICU occupancy, but also resource data such as PBB, admissions, discharges/transfers for all units and wards, and temporal information such as days, weeks and public/school holidays. One advantage of the models used was that when using scaled data, it was possible to directly understand the importance of specific features on the predictions. The predictions from the regression models relied most heavily on recent PICU-related information (figure 4) while the most important features used by the classification models were mostly temporal-related features (figure 5). In particular, whether or not it was a weekend. The models in our study also predominantly used PICU features as opposed to features from other wards, indicating that in general resource information from other wards outside of the PICU were of lesser importance for predicting future bed availability.

A main challenge encountered was that the forecasting tasks became increasingly difficult over longer forecasting horizons. This was in contrast with other similar works in literature^7^ which successfully predicted full hospital occupancy up to 60 days in the future. There are several possible explanations: (1) Our work forecasts bed availability specifically in the PICU on a daily basis, which is a challenging problem when the unit only contains 12 beds compared with a whole hospital containing hundreds of beds. (2) The random nature of unplanned admissions and discharges means there is a large amount of random variance relative to the total number of beds. This is in contrast to other studies which forecast the occupancy of beds in an entire hospital^7 19 20^ and aggregate the data into weekly totals to reduce variance.^19 20^ Aggregation into low-granularity data was judged to reduce the clinical usefulness of this work in PICU setting, and instead, other smoothing techniques were applied.

While this project uses data from PICU for critical care bed availability prediction as a case study, the methodology may be used to predict other wards and clinical areas’ bed availability. This is because the hospital bed management data, for example, the number of beds occupied and available, expected admissions and discharges, is generic data collected across all clinical areas and hospitals, regardless of the patients’ age. PICU and children’s hospitals are generally smaller in size compared with adult ICU and hospitals. Forecasting errors are generally more significant when the bed capacity of a unit is smaller. Thus, in the PICU case, forecasting was expected to be more challenging. For this reason, when generalising this method to forecast adult ICU bed availability, we would expect it to be slightly easier to get a better forecasting performance relative to the bed capacity as adult ICU usually have in excess of 12 beds.

We treated the number of beds below zero (ie, the use of unfunded beds) as the same as zero beds in our models. This was a pragmatic decision agreed between our research team and clinical management team prior to commencing this current study. This was because there was no prior study that used hospital bed management data to forecast ICU bed availability in the literature. We, therefore, wanted to simplify the methodology to successfully answer the main research question of whether ICU bed availability forecasting could be achieved using hospital bed management data and time series modelling. Now that we have answered this important question, it is possible in the future to treat the unfunded beds in the way that they are recorded in the hospital bed management data or to treat them as zero as in this current work, at the time of developing a specific model for a specific ward/unit. That should be a joint decision between the data team and the key stakeholders of the ward/unit where the specific model may be applied.

## Conclusions

Forecasting ICU bed availability is feasible using relatively simple time series models and routinely collected hospital-wide bed management data without needing individual patient-level data. Validation in a larger multicentre study is warranted.

## Data Availability

No data are available.
